# Unveiling Trophic Functions of Uncultured Protist Taxa by Incubation Experiments in the Brackish Baltic Sea

**DOI:** 10.1371/journal.pone.0041970

**Published:** 2012-07-30

**Authors:** Felix Weber, Javier del Campo, Claudia Wylezich, Ramon Massana, Klaus Jürgens

**Affiliations:** 1 Department of Biological Oceanography, Leibniz Institute for Baltic Sea Research Warnemünde, Rostock, Germany; 2 Department of Marine Biology and Oceanography, Institut de Ciències del Mar, CSIC, Barcelona, Catalonia, Spain; Université Paris Sud, France

## Abstract

**Background:**

Our knowledge of the phylogeny and diversity of aquatic protists is rapidly increasing due to molecular surveys and next-generation sequencing approaches. This has led to a considerable discrepancy between the taxa known from cultures and those known from environmental 18S rRNA gene sequences. Hence, it is generally difficult to assign ecological functions to new taxa detected by culture-independent molecular approaches.

**Methodology/Principal Findings:**

A combination of unamended dark incubations and 18S rRNA sequencing was chosen to link molecular diversity data of uncultured protists with heterotrophic, presumably bacterivorous, growth. The incubations, conducted with Baltic Sea brackish water, resulted in a consistent shift from a protistan community dominated by phototrophs to one in which heterotrophs predominated. This was determined on the basis of cell abundance and 18S rRNA sequences derived from fingerprint analysis and clone libraries. The bulk of enriched phylotypes after incubation were related to hitherto uncultured marine taxa within chrysophytes, ochrophytes, choanoflagellates, cercozoans, and picobiliphytes, mostly represented in recently established or here defined environmental clades. Their growth in the dark, together with coinciding results from studies with a similar objective, provides evidence that these uncultured taxa represent heterotrophic or mixotrophic species.

**Conclusions/Significance:**

These findings shed some light into the trophic role of diverse uncultured protists especially within functionally heterogeneous groups (e.g., chrysophytes, ochrophytes) and groups that appear to be puzzling with regard to their nutrition (picobiliphytes). Additionally, our results indicate that the heterotrophic flagellate community in the southwestern Baltic Sea is dominated by species of marine origin. The combination of unamended incubations with molecular diversity analysis is thus confirmed as a promising approach to explore the trophic mode of environmentally relevant protist taxa for which only sequence data are currently available.

## Introduction

In terms of abundance and biomass, heterotrophic protists are an essential component of planktonic communities in aquatic systems [1]. Among them, heterotrophic nanoflagellates (HNF) are the main grazers of prokaryotic cells and other picoeukaryotes [2], [3], and are able to shape bacterial community structure [4], [5]. Additionally, heterotrophic protists transfer significant amounts of bacterial production to higher trophic levels [6], and serve as important agents for nutrient remineralisation in aquatic food webs [7].

Traditional approaches such as microscopy and cultivation techniques have provided valuable data on the distribution and abundance of aquatic protists [8], [9], [10], and on the autecological properties of several cultured representatives [11], [12]. However, they have proven to be insufficient to describe the diversity and taxonomic composition of natural protistan assemblages [13]. In particular, small protists often lack distinctive morphological characteristics, making them hard to distinguish by light or electron microscopy, even at the class level [14]. Their identification is further complicated by the fact that cultivation and isolation attempts are known to be very selective for opportunistic species, which can cope better with altered *in vitro* conditions [15], [16]. Such culture conditions often involve media that differ substantially from the organisms’ aquatic surroundings, while surplus in nutrients can favor the growth of species that play only a minor role in the environment [17], [18]. Therefore, a growing consensus exists that the majority of microbial eukaryotes are yet to be cultured, and, consequently, groups without any cultured representatives may dominate in various oceanic regions [19], [20].

Just over a decade ago, molecular techniques based on the analysis of small-subunit rRNA genes began to be applied to diversity analyses of microbial eukaryotes [21], [22], [23], [24]. Nowadays, fingerprinting techniques and the construction of genetic libraries are routinely used to investigate protist communities, and they have considerably advanced our knowledge of the diversity and distribution of marine and freshwater protists [25], [26]. For example, several novel lineages within the marine stramenopiles (MAST), novel alveolates (MALV), and the recently discovered picobiliphytes, have been detected through environmental surveys [27], [28], [29], [30]. The application of extensive genetic libraries, high-throughput sequencing, and metagenomics has continued to expand the datasets of partial 18S rRNA gene sequences [26], [31], [32], [33]. Together, these comprehensive methodologies have revealed an unexpected magnitude of protist biodiversity in aquatic environments and resolved important aspects of their biogeography [34], [35], [36], [37].

Nonetheless, linking ecological functions to this diversity data is still a major challenge. This is due to the considerable discrepancy between the taxa known from cultures and those inferred from environmental 18S rRNA sequences [38]. Sometimes, environmental sequences affiliate with well-known groups and can therefore be assigned a tentative functional mode (e.g., prototroph or heterotroph). However, environmental sequencing frequently recovers phylotypes distantly related to cultured representatives, e.g., within the stramenopiles, and consequently their ecological function remains unknown [39]. Only a few studies have combined cultivation-independent techniques with approaches aimed at revealing the functional characteristics of different protists groups, such as the application of specific oligonucleotide probes for fluorescent in situ hybridization (FISH) [30], [40], sometimes in combination with bacterial uptake experiments [27], [41], [42]. Other methodologies, such as those designed to identify phagotrophic protists, include stable isotope probing [43] and sophisticated cell sorting analyses that are complemented by subsequent 18S rRNA sequencing [44].

As an alternative approach, unamended seawater incubations in the dark have been used. These stimulate the growth of heterotrophic flagellates (HF) that are abundant *in situ*, and are generally mostly uncultured, but does not trigger the mass growth of typical cultivable flagellates [45], [46]. Subsequent 18S rRNA analysis in these incubation experiments have allowed the identification of otherwise undetected protistan taxa [47], [48].

In the present study we followed the approach of unamended dark incubations for the first time in a more nutrient rich and brackish water environment in order to detect those uncultured protists that exhibit a heterotrophic life style. Thereby, we succeeded in preferentially enriching uncultured heterotrophic flagellates, while minimizing the culturing bias [46]. Subsequent 18S rRNA analysis revealed that most of the taxa at the end of the incubations were only distantly related to cultured representatives. Unexpectedly, most of the enriched phylotypes of this brackish water site showed highest similarities to environmental clones of marine origin. Overall, our study provides valuable information on the heterotrophic protist community of the Baltic Sea.

## Results

### Microbial Successions

**Figure 1:**
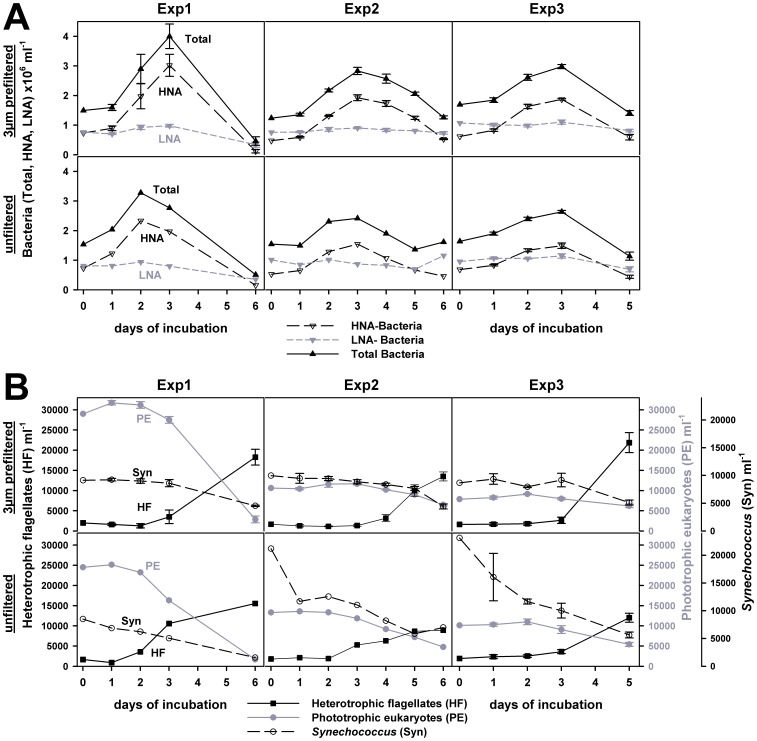
Cell number development in the course of three incubation experiments. (A) the abundance of total, high, and low nucleic acid containing bacteria (total, HNA, and LNA) for the 3-µm filtered and unfiltered treatments, respectively. (B) Cell numbers of *Synechococcus* (Syn), phototrophic eukaryotes (PE), and heterotrophic flagellates (HF) in the 3-µm filtered and unfiltered treatments. Error bars indicate the standard deviation in triplicate incubation bottles.

The development of bacteria, *Synechococcus*, phototrophic eukaryotes, and HF showed a consistent pattern in the three incubation experiments, with a remarkably low variance within the triplicates (Fig. 1). Total bacterial abundance increased to form a peak after about 3 days, with cell numbers decreasing after 5–6 days to values similar to or below the initial ones. Changes in bacterial abundance were due to the rapid response and subsequent decline of high nucleic acid containing bacteria (HNA) whereas low nucleic acid containing bacteria (LNA) remained relatively constant throughout the experiments (Fig. 1A). Additional experiments revealed that leucine incorporation, as a measure of bacterial production, also increased considerably during the incubations, reaching a maximum one day before the peak in bacterial abundance (Weber *et al.*, unpublished data). Coincidently with the decrease of bacteria, HF increased significantly and were enriched 5- to 13-fold at the end of the incubation. The cell numbers of HF detected in unfiltered treatments were generally lower (1,300–15,000 cells ml^−1^) than those enumerated in the 3-µm filtered samples (1,200–21,000 cells ml^−1^). Additively, larger forms of HF (>3 µm) as well as acanthoecid choanoflagellates (with silicious loricae) were observed primarily in the unfiltered treatments. Phototrophic eukaryotes were initially 5–15 times more abundant than HF but began to decline after 2–3 days of incubation such that by the end of the experiments their cell numbers were lower than those of HF (Fig. 1B).

### Compositional Shift Towards Heterotrophic Taxa Revealed by DGGE

**Figure 2:**
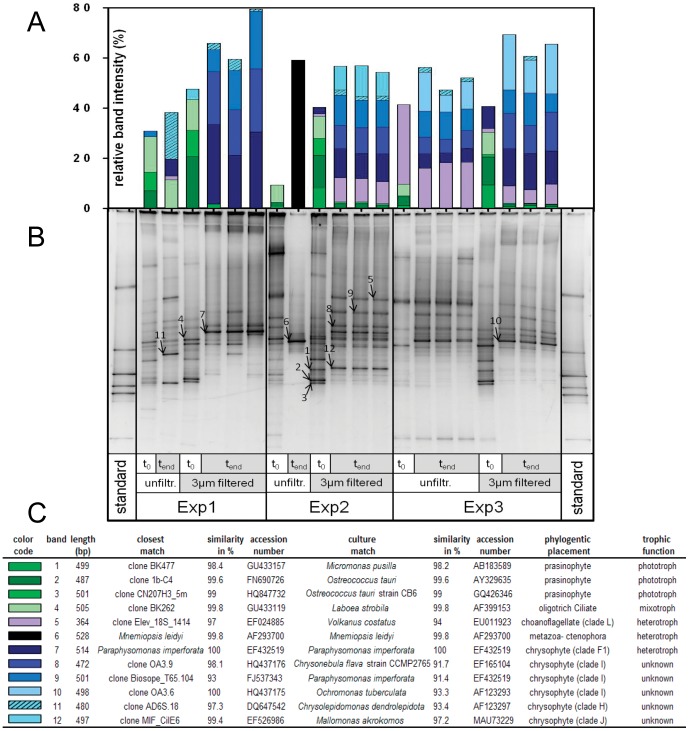
Comparison of 18S rRNA fingerprints at the start and end of three incubation experiments. (A) Stacked bar chart showing the relative intensity of the sequenced gel bands in each lane from filtered and unfiltered samples taken at the start and end of the incubation within the three experiments. Each color of the stacked bars represents one band and the length corresponds to the relative band intensity. (B) Numbered arrows indicate sequenced bands in the inverted DGGE image. (C) Information on the phylogenetic affiliation and trophic functions of sequenced bands with the band numbers referring to the bands in the DGGE image and the color code referring to the stacked bars chart.

Both molecular techniques, i.e., DGGE and clone libraries, were RNA-based in order to principally detect actively growing protists. The DGGE fingerprints of 18S rRNA obtained at the start (t_0_) and the end (t_end_) of incubation revealed compositional shifts in the protist community, again with very low variance in the triplicates (Fig. 2, S1).

The presence or absence of bands at a certain position was used to calculate the similarity of all samples, which was then displayed in a multidimensional scaling plot (Fig. S1). The 3-µm filtration step caused only minor changes in community structure whereas the incubation process was critical, since samples of the same time point were more similar than those obtained from different time points (Fig. S1). This finding was consistent across all three experiments and in triplicate samples, with two exceptions: the unfiltered t_end_ sample of Exp1 (black open triangle) was very similar to the respective t_0_ sample (black open square), and the unfiltered t_end_ sample of Exp2 (dark gray open triangle) was an outlier to all other samples due to the presence of the ctenophore *Mnemiopsis leidyi*, represented by one very dominant DGGE band (band 6, Fig. 2).

By quantifying the relative intensities of the sequenced bands in each DGGE lane we obtained a first impression of the taxa contributing to the community shift during the incubation (Fig. 2). Among the dominant bands in the t_0_ samples were three belonging to the phototrophic prasinophytes *Micromonas pusilla* (band 1) and *Ostrecoccus tauri* (bands 2 and 3), and one belonging to the mixotrophic oligotrich ciliate *Laboea strobila* (band 4). Together, these four bands accounted for 10–30% of the relative band intensity in unfiltered treatments and 30–45% in the 3-µm filtered treatments of the t_0_ samples. The same bands were absent or contributed <3% in most of the t_end_ samples (except for the unfiltered t_end_ sample of Exp1, in which *Laboea strobila* still accounted for 10% of the relative band intensity).

In contrast, bands representing known heterotrophic taxa such as *Paraphysomonas imperforata* (band 7) or an uncultured choanoflagellate (band 5) as well as various bands belonging to uncultured chrysophytes (bands 8–12) of unknown trophic function seemed to benefit from the incubation, as they were of low signal intensity or undetected in the t_0_ samples but became dominant by the end of the incubation. For instance, all bands affiliated with uncultured chrysophytes (related to environmental clade I) contributed a maximum of 2% to the relative band intensity in the t_0_ samples and 20–50% to that of the 3-µm filtered samples processed at the end of the incubation (Fig. 2).

### Compositional Shifts Towards Heterotrophic Taxa Revealed by Clone Libraries

**Figure 3:**
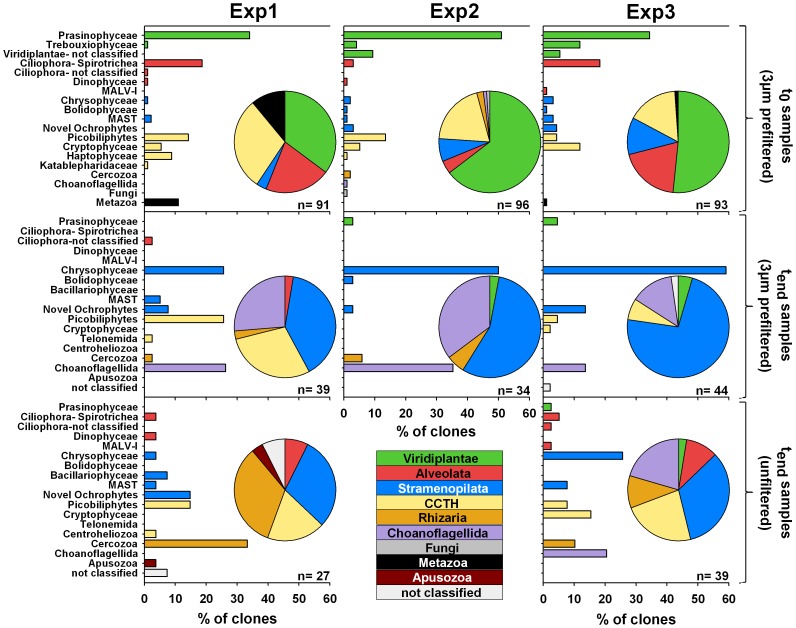
Phylogenetic composition in clone libraries constructed from the three incubation experiments. Upper and middle panels show the composition of clones in the 3-µm filtered initial and final incubation samples, respectively. The unfiltered final incubation samples are represented by the lower panels. For each library, the proportion of clones within the major taxonomic groups is displayed as pie charts. A finer resolution of the taxonomic groups and their contribution to each library is shown in the bar charts.

To achieve a finer resolution and gain more valuable phylogenetic information on the protistan community composition at the start and end of the incubation, 18S rRNA clone libraries were prepared. In total, eight clone libraries from the three experiments were constructed; resulting in 269 and 182 analyzed clones for t_0_ and t_end_ samples, respectively (Fig. 3). In the three initial (t_0_) clone libraries from the 3-µm filtered samples, Viridiplantae sequences were dominant (35%, 52% and 65%, respectively), with those of the prasinophytes *Ostreococcus tauri*, *Ostreococcus lucimarinus,* and *Micromonas pusilla* representing the largest fraction. Among the CCTH group (including cryptomonads, centrohelids, telonemids, haptophytes and picobiliphytes, as defined by Burki *et al*. [49]), which accounted for 16%, 20%, and 30% in the t_0_ libraries, half of the sequences belonged to cultured microalgal species within the cryptophytes and haptophytes while the other half of the sequences originated from yet uncultured picobiliphytes. Alveolate sequences mostly belonged to spirotrich ciliates related to *Laboea strobila* and *Strombidium biarmatum* and other spirotrichs that could not be further classified. Whereas only a single choanoflagellate-related sequence was found in the three t_0_ samples, choanoflagellate-related sequences accounted for 14–35% in the t_end_ samples, except for the unfiltered sample of Exp1, in which no choanoflagellates were found. The contribution of cercozoans to the protistan assemblage was low in the t_0_ samples but increased especially in the unfiltered samples to as much as 10–33% by the end of incubation. Stramenopiles comprised up to 12% of the clones in the t_0_ libraries and 30–73% of those in the final samples. This change was mainly driven by a vast increase of sequences related to chrysophytes and novel ochrophytes.

### Assignment of Trophic Functions

**Figure 4:**
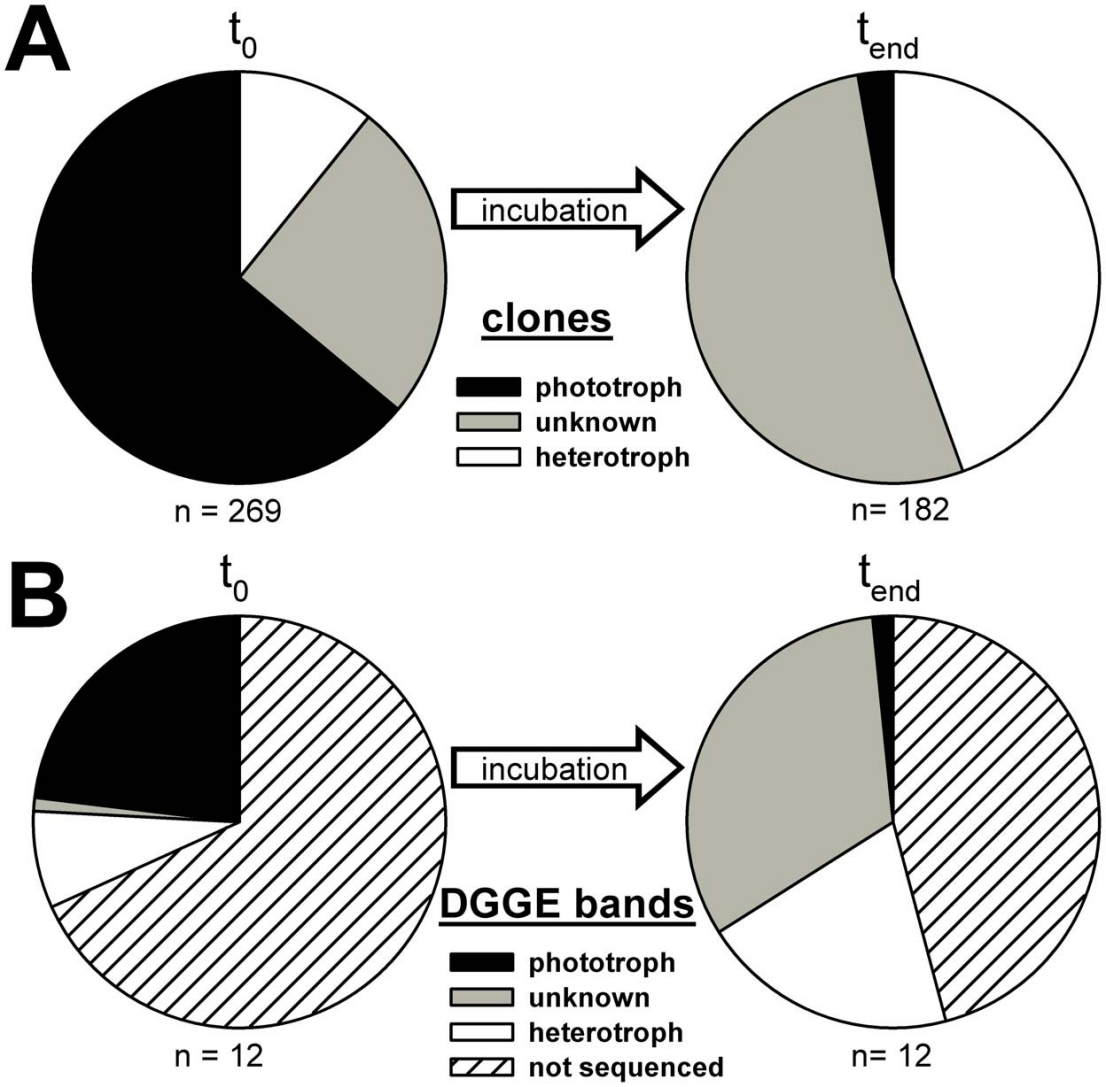
Community shift towards heterotrophic taxa revealed by assignment of trophic functions to 18S rRNA sequences. The proportion of sequences assigned a trophic function (phototroph, heterotroph, unknown) at the start and end of incubation, as determined in clone libraries (A) and DGGE analysis (B).

To assign a potential trophic function to the detected sequences, and particularly to distinguish photo- and heterotrophs, we searched for the closest cultured representatives in GenBank via BLAST. A similarity value ≥98% was used as the basis for attributing the reported trophic function (phototroph or heterotroph) of cultured species to the sequence. Irrespective of this similarity value to the closest cultured representative, all sequences closely affiliated with choanoflagellates, cercozoans (excluding sequences affiliated with chlorarachneans), and to members of the MAST cluster were assumed to be heterotrophic. All other sequences belonging to different phylogenetic groups and not complying with the similarity criterion were classified as unknown in their trophic function. This classification revealed a clear shift from phototrophic to heterotrophic species during the incubation, as evidenced by sequences derived from the clone libraries (Fig. 4A) and from excised DGGE bands (Fig. 4B). In clone libraries, sequences related to phototrophs decreased from 64% to 3% of all clones whereas sequences related to heterotrophs increased from 11% to 44%. The fraction of protists with an unassigned trophic function increased from 25% to 53%. The bulk of these unassigned sequences belonged to uncultured chrysophytes, novel ochrophytes, and picobiliphytes. A similar pattern was observed in the DGGE band analysis.

### Phylogenetic Affiliation of Enriched Protist Taxa

**Figure 5:**
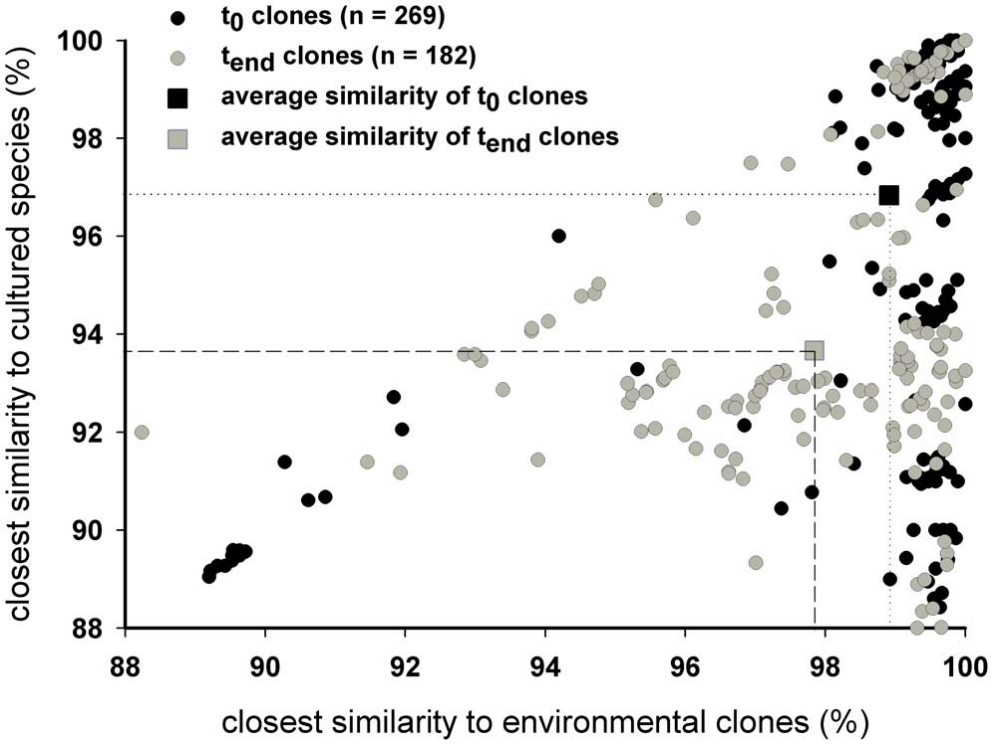
Novelty pattern determined for all clones obtained at the start and end of incubation experiments. Each circle represents one clone and its percent similarity to the closest environmental clone and the closest cultured species in GenBank. The mean similarity to both is indicated by the squares.

BLAST searches for the closest cultured representative and the closest environmental sequence in GenBank resulted in two similarity values for each clone. These were represented in a scatter plot displaying distinct novelty patterns for clones obtained before and after the incubation (Fig. 5). Clones of the t_end_ samples were less similar to cultured representatives than t_0_ clones (average similarities: t_0_ = 96.5%; t_end_ = 93.6%). Additionally, there was a slight trend among clones at the end of the incubation to be less similar to environmental sequences than clones prior to incubation (average similarities: t_0_ = 98,9%; t_end_ = 97,8%).

**Figure 6:**
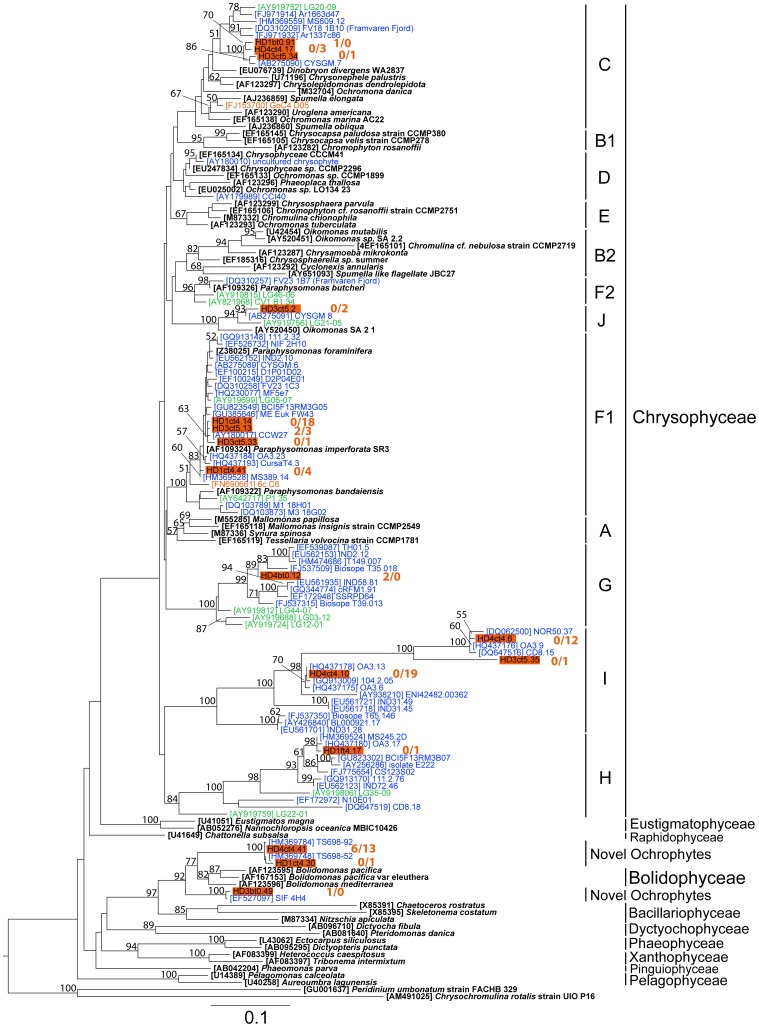
Phylogenetic affiliation of clones within ochrophytes. Maximum-likelihood phylogenetic tree constructed with 142 partial and complete ochrophyte 18S rRNA sequences (962 informative positions). Bootstrap values >50% are displayed. Clades within the chrysophytes follow the notation of del Campo and Massana [38]. Sequences indicated by red bars originate from this study; the numbers refer to the found clones in t_0_ and t_end_ samples, respectively. Sequences from cultured representatives are shown in italics. Environmental clones are highlighted, according to their origin, in blue for marine clones and green for freshwater clones. The scale bar indicates 0.1 substitutions per position.

**Figure 7:**
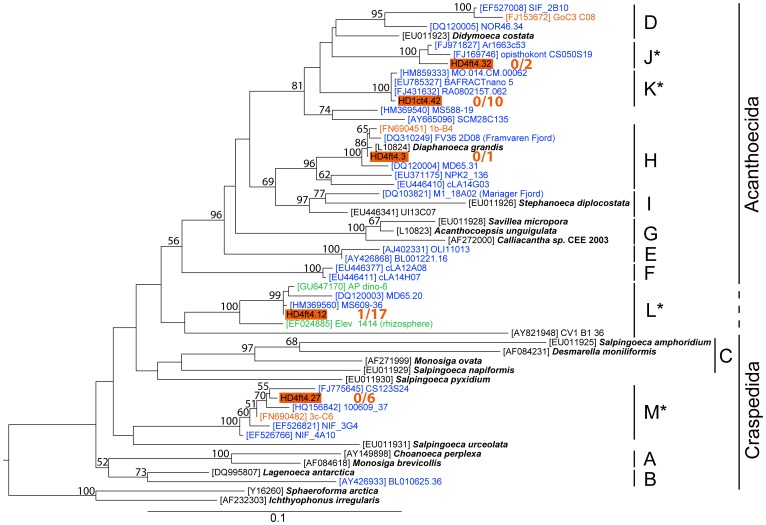
Phylogenetic affiliation of clones within choanoflagellates. Maximum-likelihood phylogenetic tree constructed with 54 partial and complete choanoflagellate sequences (733 informative positions). Clades A–I follow the notation of del Campo and Massana [38]. Four additional clades (J–M) are introduced. For further description see legend of Figure 6.

**Figure 8:**
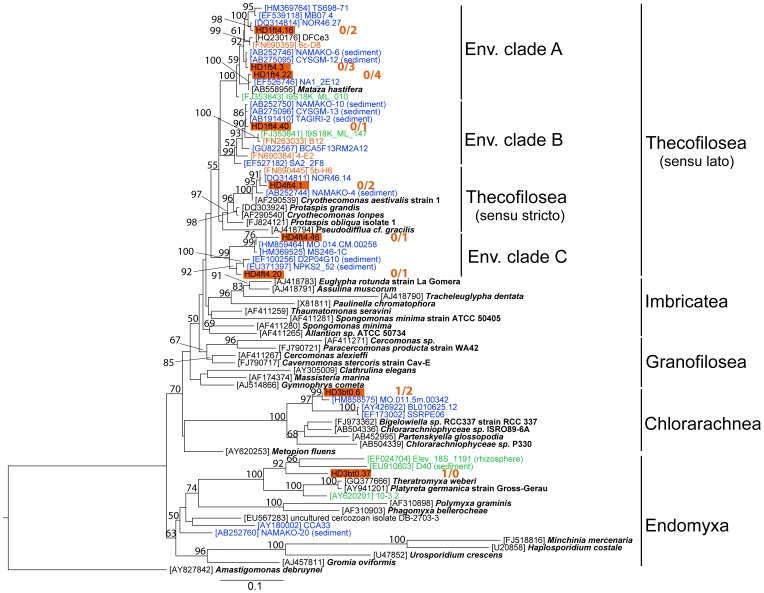
Phylogenetic affiliation of clones within cercozoans. Maximum-likelihood phylogenetic tree constructed with 77 partial and complete cercozoan sequences (1059 informative positions). For further description see legend of Figure 6.

To identify the phylogenetic affiliation of sequences representing taxa seemingly able to grow in the dark, we constructed phylogenetic trees for ochrophytes, choanoflagellates, cercozoans, and picobiliphytes. Within these groups most of our sequences were rather closely related to clones of marine origin than to clones of freshwater or brackish water systems (Fig. 6, 7, 8, S2). The bulk of the ochrophyte sequences affiliated with chrysophytes. The topology of the latter in the phylogenetic tree (Fig. 6) was in accordance with the branching order reported by del Campo and Massana [38]. Less than half of the chrysophyte sequences belonged to clades (F1, C, J) containing both, cultured representatives of the genera *Paraphysomonas, Dinobryon, Spumella* and *Oikomonas*, and environmental freshwater and marine sequences. Rather, most of the chrysophyte sequences affiliated with clades that exclusively consisted of environmental sequences, as determined based on good bootstrap support (>84%). Some sequences fell into clades G and H, related to sequences originating from various marine and freshwater habitats, while the majority appeared in clade I, presumably an exclusively marine cluster [38]. Within clade I, 19 and 12 sequences clustered at two distinct positions, respectively, building a long branch with sequences from the North Atlantic (clone 104.2.05) and the Arctic Ocean (clone NOR50.37). Furthermore, clade I included sequences derived from a HF peak determined in two other unamended dark incubation experiments that had been carried out with water samples collected in the Mediterranean Sea [38] and the Norwegian Sea [50]. Another large fraction of sequences (20 clones) grouped in novel ochrophyte clades distantly related (94% on average) to *Bolidomonas* species and closely related to heterotrophic protists sorted by flow cytometry (clones TS698-92 and TS698-52 [44]).

The choanoflagellate tree (Fig. 7) recovers nine clades (A–I) as defined by del Campo and Massana [38] and four additional clades (J–M) as defined by the present study. All clades comprise solely environmental sequences, including those from our experiments. Only one choanoflagellate clone (HD4ft4.3) was closely affiliated with a cultured organism (*Diaphanoeca grandis*), in clade H. The remaining 36 clones clustered within the four novel clades, mostly within clades K and L. Whereas clades J and K contain sequences of marine origin only, clade L also comprises sequences from freshwater and soil habitats. In addition to marine sequences, clades H and M contain sequences from the Baltic Sea ice (clone 3c-C6) and wintertime water (clone 1b-B4) of the Bothnian Sea and Gulf of Finland.

The bulk of cercozoan-related sequences branches off within Thecofilosea *sensu lato*, made up of Thecofilosea *sensu stricto* and the environmental clades A (defined by Yabuki and Ishida [51]), B, and C (defined herein) (Fig. 8). All sequences related to Thecofilosea *sensu lato* were detected in t_end_ samples, and preferentially in the unfiltered treatments. Most of our sequences belonged to environmental clade A, represented by three OTUs related (96–99% similarity) to the recently described organism *Mataza hastifera* [51]. The environmental sequences within clades A and B derive from a variety of systems, including marine, freshwater, and brackish water sites (Baltic Sea). However, our sequences showed higher similarity to marine representatives. Clade C solely contains sequences of marine origin. The Thecofilosea *sensu stricto* contains one OTU that is closely affiliated (98%) to Baltic Sea and marine clones and to *Cryothecomonas aestivalis* strain 1. Among the marine sequences within Thecofilosea *sensu lato*, several were detected in sediment samples. Sequences within the Chlorarachnea and Endomyxa were related to either solely marine or solely freshwater clones, respectively.

Among marine picobiliphytes (Fig. S2), 49 sequences (30 originating from the t_0_ samples and 19 from the t_end_ samples) affiliated exclusively with clones of clade BP1, as defined by Cuvelier *et al*. [52]. The effect of growth during incubation probably only applied to the OTU HD1ft4.11, represented by four clones in the t_0_ sample and by 11 clones in the t_end_ sample. Furthermore, clade BP1 also contains sequences representing putatively heterotrophic taxa, as identified by Heywood *et al*. [44].

## Discussion

In this study, the composition of heterotrophic protists in Baltic Sea surface water was analyzed through an approach based on unamended dark incubations. These were used to create a “functional filter” able to promote a slight enrichment of heterotrophic (bacterivorous) flagellates due to a stimulation of the naturally occurring bacterial assemblage. The growth of heterotrophic bacteria was driven by HNA-containing bacteria, thought to represent the active bacterial community and responsible for increases in bacterial production [53]. The maximum bacterial abundance in the experiments was in the range of 2.5−4×10^6^ cells ml^−1^, consistent with that commonly reported for the Baltic Sea during summer/autumn phytoplankton blooms [54], [55], [56]. We assume that the dark bottle incubations induced a moderate nutrient pulse, with the bulk of organic matter likely deriving from algal degradation–comparable to productive periods in the Baltic Sea.

The development of HF in the course of the incubations was obviously linked to an increase in bacterial abundance. Although the 3-µm filtered and unfiltered treatments showed a similar succession, the growth of HF was more pronounced in the former. In the unfiltered treatments, interactions of HF with larger protists probably became important, evident from the increased contribution of ciliate sequences to the clone libraries. Ciliates can be considered to affect HF abundance by top-down control [6], or simply by prey exhaustion when they act as competitors for bacterial prey. Another group of protists that was predominantly represented in clone libraries of the unfiltered samples consisted of the Thecofilosea-related cercozoans, which might have been omnivores and were thus preying also on HF.

Even though primer systems targeting different regions of the 18S rRNA were used in the DGGE and clone libraries, the two techniques identified similar phylotypes contributing to the protistan community shift. This was particularly true for chrysophytes clade I and choanoflagellates clade L, which constituted a considerable proportion of the protist community at the end of the incubations. This latter point indicates that both groups profited from the bacterial stimulation and are thus very likely bacterivorous. In general, many of the phylotypes in the incubated samples were characterized by an elevated degree of novelty with respect to cultured representatives and even to environmental records (Fig. 5). In the initial samples, many of these novel sequence types were absent, while others occurred at low clonal abundance. In these samples, they were largely masked by the presence of numerous well-known phototrophic protists in the clone libraries. In contrast, the higher number of novel sequences (mostly chrysophytes, ochrophytes, choanoflagellates, cercozoans and picobiliphytes) at the end of the incubation allowed their detection within the clone libraries. As reported from other studies using bottle incubations, our experiment also enabled the identification of protistan taxa that otherwise might have been undetected [47], [48].

It has long been pointed out by microbial ecologists that experiments which include containment of water samples may influence cell concentrations and the activity of microbial cells [57], [58]. Moreover, changes in the bacterial and protistan community structure after one to three days of enclosure have been reported [48], [59]. These multiple impacts on microbial assemblages under confined conditions have been compiled in the literature as the so called “bottle effect”. Currently there is no consensus whether the bottle size, the volume of enclosed water and the resulting surface to volume ratio has an influence on the outcome of bottle effects [60]. In our study we observed shifts in the protistan community structure (from phototrophs to heterotrophs) as a consequence of the experimental manipulation (incubation in the dark). Although we cannot preclude container effects during our incubation and do not exactly know to what extend our results reflect the *in situ* diversity of the heterotrophic protist community, we think that these incubations enable to study an important portion of this community which is generally hidden by most other approaches.

Several methodological limitations, specifically during nucleic acid extraction and PCR amplification, may block the possibility of an absolute and detailed quantification of certain taxa based on their clonal abundance [61]. Nevertheless, by comparing the communities at the start and end of the incubation we obtained relative estimates of the taxa that respectively grew or declined during the course of the experiment. Moreover, RNA-based approaches, as were employed here, are known to provide a more accurate picture of community structure than DNA-based approaches since the latter can suffer from the taxon-specific rDNA copy number and the amplification of DNA derived from inactive and dead cells or even extracellular genetic matter [26], [62].

### Does a Culturing Bias Apply to Unamended Dark Incubations?

Seawater enclosures, sometimes in combination with size fractionation, have a long tradition of use in ecological studies. They have contributed greatly to our understanding of carbon fluxes in the oceans, trophic interactions in general, and the regulatory mechanisms of bacterial biomass distribution by either nutrient supply or protist consumption [58], [63], [64], [65]. They have also served as precursors for isolation attempts aimed at studying and describing a single microbial species obtained in pure culture [9], [66]. On the other hand, the selectivity of this approach towards opportunistic species has led to misinterpretations of the quantitative contribution and widespread dispersal of various protist groups [17], [18]. This issue was addressed in two recent studies that investigated the effect of nutrient supply in incubation experiments. Both revealed increasingly rapid and pronounced community shifts as the incubation medium became richer and more complex [48], [46]. Specifically, whereas unamended incubations promoted the growth of uncultured HF, these organisms were displaced towards cultured representatives in incubations altered by allochthonous nutrient addition [38], [45].

In our experiments a considerable proportion of clones (12% of all clones) at the end of the incubation were closely affiliated with *Paraphysomonas imperforata*. This could indicate a culturing artifact, such as shown by Lim *et al*. [18] for Atlantic seawater incubations with substrate addition. However, based on the occurrence of sequences related to *P. imperforata* in both the DGGE and the clone libraries prior to the incubation, we conclude that this organism was a naturally dominant member of the protist community at the sampling site and remained so throughout the incubation. In addition, during our sampling campaign in Heiligendamm, well known and easily cultivable species like *Cafeteria roenbergensis* and various *Bodo* species were frequently isolated and subsequently maintained in wheat grain cultures (F. Weber and A.P. Mylnikov, unpublished data) but none of these flagellate sequences were detected in our unamended incubation experiments.

Another line of evidence that speaks against a culturing bias is the fact that most of the sequences at the end of the incubation belonged to taxa that were only distantly related to cultured representatives (Fig. 5), some of which were already present in the initial samples. A culturing artifact would have yielded sequences closely affiliated with well known cultured representatives, which are only seldom found in natural samples [46]). With regard to that our findings are more consistent with those obtained by culturing-independent environmental approaches. This allows the conclusion that culturing bias can be disregarded as the main explanation for our results.

### Novel Heterotrophic and Putatively Heterotrophic Taxa

Similar to other dark incubations [45], [46], [67], the experimental design of our study promoted the growth of HF while suppressing that of phototrophic species. Furthermore, based on the tightly coupled bacteria and HF successions, a strong potential for bacterivory can be assumed. This enabled us to link the phylogenetic data obtained at the end of the incubation with the presence of a heterotrophic, phagotrophic life style.

The proportion of sequences unambiguously assigned an obligate heterotrophic function increased during the incubation from 11% to 44% of all found clones. One reason for that was the large contribution of sequences closely related to the bacteria-consuming species *P. imperforata.* Another reason why a heterotrophic function could be inferred for various other clones indeed distantly related to cultured organisms was their phylogenetic placement within obligate heterotrophic protist groups such as the choanoflagellates [68] and cercozoans (excluding chlorarachneans).

Interestingly, the relative abundance of sequences assigned an unknown trophic function doubled during the dark incubation, indicating that at least some should be regarded as facultative heterotrophic flagellates. Prime examples are two OTUs within environmental clade I of chrysophytes, both of which had increased in clonal abundance by the end of the incubation (HD4ct4.10 and HD4ct4.6, represented by 19 and 12 clones, respectively). This clade has been shown to comprise pigmented representatives (clone Biosope_T65.146 [19]) as well as putative heterotrophic flagellates (clone CD8.15 [45]; clones OA3.9 and OA3.13, [46] that grew in samples collected from two distinct oceanic regions and then subjected to unamended dark incubation. Our clones are distantly related to the ones inferred from pigmented cells but very closely affiliated with the sequences deriving from putative heterotrophic flagellates. Therefore, the fact that virtually the same taxa have been detected in three independent studies using the same technique to target heterotrophic and bacterivorous protists provides strong evidence that these chrysophytes of environmental clade I are heterotrophic or mixotrophic flagellates.

Another set of clones was designated as novel ochrophytes according to their phylogenetic placement next to well known photosynthetic ochrophyte groups [69] such as bacillariophytes and raphidophytes. These sequences were represented by three OTUs having sister group relationships to bolidophytes, a group of photosynthetic flagellates that putatively contain mixotrophic members [43]. Similar to the other groups discussed herein, certain OTUs (HD4ct4.41 and HD1ct4.30) contributed more to clone libraries prepared at the end of the incubation than to those derived from the initial samples. Moreover, the closest relatives (clones TS698-92 and TS698-52) of these OTUs were reported from a study in which heterotrophic eukaryotes were sorted by the presence of lysotracker fluorescence and the absence of chlorophyll fluorescence before they were characterized with respect to their 18S rRNA genes [44]. Additionally, a closely (99% similarity) related sequence (clone BLACKSEA_cl_48, HM749950, not shown in the tree) was recently detected by an rRNA-based study of the non-illuminated depth of the Black Sea redoxcline [70]. It therefore seems likely that these novel ochrophyte species have a phagotrophic potential.

Another group of uncultured protists that apparently grew in our experiment consisted of picobiliphytes, which represent a possible first-rank taxon phylogenetically related to cryptophytes and katablepharids [30], [52]. Based on microscopy observations in combination with specific 18S rRNA FISH probes, picobiliphytes were suggested to be a new plastid-bearing algal group [30], [52]. Evidence to the contrary is that picobiliphytes represented a significant fraction of single amplified genomes (SAG)–determined subsequent to flow cytometric cell sorting of heterotrophic eukaryotes. [44]. Furthermore, the results of whole-genome shotgun sequencing of one representative from each of the three picobiliphyte sub-clusters suggested that they are heterotrophic because no indication for plastid DNA or nuclear-encoded plastid-targeted proteins could be found [71]. The growth of picobiliphytes (especially OTU HD1ft4.11) in our unamended dark incubations supports the postulated heterotrophy of these organisms from another perspective.

### Which Taxa are Likely to Constitute the Assemblage of Heterotrophic Protists at the Sampling Site?

Our study represents only a spatial and temporal snapshot of the protistan assemblage in the southwestern Baltic Sea. Nonetheless, it offers an impression of the taxa that presumably play an important role as heterotrophic and bacterivorous protists at this coastal site. Indeed, taxa capable of growth in dark incubations likely belong to flagellate groups able to control and rapidly react to sporadic bacterial bursts, thus providing insight into the heterotrophic protist community during naturally occurring pulses of bacterial food supply. In the Baltic Sea, these pulses are typically observed after phytoplankton blooms. However, frequent fluctuations in the protistan community composition can be expected, particularly for coastal areas in the southern Baltic Sea, due to seasonal variations and to transient perturbation events (mixing, upwelling, riverine influx, salinity changes) [42]. Hence, this may explain why the phylotypes found in this study are not in general agreement with those in the study of Piwosz and Pernthaler [42], carried out in the Gulf of Gdansk, Southern Baltic Sea. Only a more frequent sampling can resolve whether this discrepancy can be attributed to the lower salinities of their study site (6–7 PSU in the Gulf of Gdansk vs. 13–16 PSU in Heiligendamm), to seasonal variations, to sporadic events, or to a combination of all three factors.

As revealed by phylogenetic analysis and BLAST searches, most of the closest relatives to our OTUs originate from marine systems and only very few from freshwater or brackish water habitats (see Fig. 6, 7, 8, S2). A similar pattern was seen in the clone libraries obtained from the Gulf of Gdansk [42]. However, the typical representatives within MAST and MALV, frequently reported in various marine studies, rarely occurred in our samples. Therefore, most phylotypes shown in our experiment to be actively growing, including those within chrysophytes, novel ochrophytes, choanoflagellates, cercozoans, and picobiliphytes, apparently represent euryhaline taxa that tolerate salinities ranging from fully marine to brackish water conditions. Further analysis employing newly designed oligonucleotide probes for fluorescent in situ hybridization will resolve the *in situ* abundance, dispersal within the salinity gradient of the Baltic Sea and seasonal fluctuations of the respective taxa.

### Conclusions

Unamended dark incubation caused two effects in our experiments: (1) a shift towards a community made up of heterotrophic protists and (2) the enrichment of primarily uncultured taxa, whose presence may have otherwise been obscured by the high abundances of phototrophs. Bringing both observations together, unamended dark incubations appear to be a powerful tool to bridge the gap between our understandings of natural protistan assemblages provided by culturing and environmental sequencing surveys. Indeed, these incubations level out the major drawbacks of both approaches, i.e., culturing bias and the loss of functional information. Additionally, our results suggest that the heterotrophic protist community in the southwestern Baltic Sea is constituted by a large proportion of as yet uncultured marine flagellates, including some that thus far have been only rarely detected, alongside with well known cultured representatives (e.g., *Paraphysomonas imperforata*). Our study therefore provides important insights into both the trophic function of various uncultured protists, and their dispersal capacity with respect to salinity.

## Materials and Methods

### Sampling

Sampling was conducted at three different dates in autumn 2008 (October 28; November 11; November 18) at the coastal monitoring station of Heiligendamm, located in the Mecklenburg-bight of the southwestern Baltic Sea (54°08,55′ N; 11°50,60′ E). Surface water samples were lifted in a bucket from a sea bridge 200 m off the sandy littoral, filtered through a 200-µm nylon mesh, and collected in sterile plastic containers that were transferred within 1 h to climate chambers. Temperature and salinity were measured during sampling by a portable conductivity meter (Cond 1970i, WTW GmbH, Weilheim Germany).

### Seawater Incubation and Experimental Setup

All plastic containers and glass bottles used during sampling and incubation were washed with 10% HCl and with water purified using the Milli-Q lab water system (Millipore), then rinsed with the seawater fraction used for the incubation. Prior to incubation, one fraction of seawater was filtered through a 3-µm polycarbonate filter (Whatman GmbH) under pressure so gentle that it did not register on the barometer attached to the vacuum pump. Both fractions of seawater (<200 µm and <3 µm) were partitioned into triplicate 1 L glass bottles. Those bottles used in the immediate analysis of the starting parameters (t_0_) were filled in the same manner but not as triplicates. All filtration steps and transfers to other receptacles were done conservatively through a tubing connection to prevent damage to fragile organisms in the samples. Incubation was carried out in the dark under near in situ temperature conditions in a 10°C climate chamber.

### Cell Numbers in the Course of Incubation

The development of heterotrophic flagellates (HF), bacteria, *Synechococcus*, and phototrophic eukaryotes (nano- and picoeukaryotes) in the incubation bottles was monitored by an almost daily subsampling. Subsamples (30–50 ml) for epifluorescence microscopy were fixed with glutaraldehyde (1% final concentration), stained with DAPI, and filtered on 0.2- and 0.8-µm pore size black polycarbonate filters (Whatman GmbH). HF enumeration was carried out with an epifluorescence microscope (Axio Imager.M1, Zeiss). For bacterial determinations, *Synechococcus* and phototrophic eukaryotes subsamples (4 ml) were fixed with paraformaldehyde and glutaraldehyde (final concentration 1% and 0.05%, respectively), deep-frozen in liquid nitrogen, and analyzed by flow cytometry [72].

### RNA Extraction and cDNA Generation

Cell material from water samples (200–350 ml) of the start (t_0_) and the end (t_end_) of the incubation was collected on 0.2-µm pore size polycarbonate filters (Durapore, Millipore). The filters were shock frozen in liquid nitrogen and stored at −80°C for subsequent extraction of nucleic acids.

RNA was extracted according to Weinbauer et al. [73] using an acidic extraction buffer. The washed and dissolved RNA was quality-checked on an agarose gel and quantified using a NanoDrop ND-1000 UV/Vis spectrophotometer (NanoDrop, Thermo Fisher Scientific). Residual environmental DNA was eliminated in RNA extracts by DNase I digestion (DNA-free-kit, Ambion) for 75 min at 37°C. Thereby, enzymatic degradation of RNA was prevented by treating each sample with 0.9 µl of an RNase inhibitor (Peqlab) per 20 µl of RNA sample. Complete removal of the DNA in each of the RNA extracts was verified by PCR, as described in the next section, using the RNA extracts as template. In all cases, amplification was negative (results not shown). To generate cDNA, 200 ng of template RNA was reverse transcribed at 42°C using the iScript Select cDNA synthesis kit (Bio-Rad) following the manufacturer’s recommendations. In addition to the random primer provided with the kit, the eukaryote-specific primer Euk B [74] was used. In each reverse transcription reaction, some of the RNA samples used as controls in the PCR were not supplemented with reverse transcriptase, in order to rule out DNA contamination.

### Denaturing Gradient Gel Electrophoresis (DGGE)

The well established eukaryote specific primers EukA and Euk516r-GC [21], [74], [75] were used to amplify the 18S rRNA fragments by PCR. The PCR mixture (50 µl) was composed of 200 µM of each deoxynucleoside triphosphate, 1.5 mM MgCl_2_, 0.3 mM of each primer, and 1.25 U of *Taq* DNA polymerase (Fermentas). PCR was started with an initial denaturation at 94°C for 2 min, followed by 28 cycles of 30 s at 94°C, 45 s at 56°C, and 2 min at 72°C. Final extension was done at 72°C for 6 min. DGGE was performed with a vertical electrophoresis system (PhorU, Ingeny). Two previously prepared stock solutions were used to cast a 6% (wt/vol) polyacrylamide gel (ratio of acrylamide to bisacrylamide 37.5∶1) with a linear gradient of denaturing conditions ranging from 25 to 55%. After loading equivalent amounts of PCR product in each lane, the gel was run at 100 V for 16 h at 60°C submerged in 0.5× TAE buffer, followed by staining with SybrGold for 1 h in the dark and visualization under UV radiation in a gel documentation system (Geldoc, BioRad). The resulting image was analyzed with the software GelCompare II (Applied Maths) in order to assign all detected DGGE bands to a certain position in the gel and to quantify their relative intensity in each lane. A 0–1 matrix was constructed based on the presence and absence of bands at a certain position and then used to create a non-metric, multidimensional scaling (MDS) ordination plot with respect to the calculated Bray-Curtis similarity coefficients using PRIMER (v6). Representative DGGE bands excised from the gel and eluted overnight at 4°C in 50 µl of nuclease-free water were stored at −20°C. To reamplify the bands, 1 µl of the eluate served as template in a PCR run under the above-described conditions, except that the reverse primer lacked the GC clamp. In most cases, bands assigned to a certain position in the gel were excised and sequenced from separate lanes to verify whether they belonged to the same taxon.

### Clone Library Construction

In total, eight clone libraries from three incubation experiments (Exp1, Exp2, and Exp3) were constructed prior to incubation (t_0_) and at the time of enriched flagellate abundance (t_end_) with samples from the 3-µm and 200-µm filtered treatments. Due to the contamination of *Mnemiopsis leidyi* in Exp2, we were able to obtain only two libraries at the end of the incubation from the unfiltered treatments.

Reverse transcribed 18S rRNA was used as template in PCRs with the primer pair Euk528F [76] and 18S-1630Rev [70], amplifying fragments of almost 1,000 base pairs. The PCR mixture was heated to 95°C for 10 min, and the target then amplified within 30 cycles of 95°C for 30 s, three different annealing temperatures (52°C, 55°C, and 58°C) applied in single reactions for 1 min followed by 70°C for 2 min, and a final extension of 10 min at 72°C. PCR products treated with the three different annealing temperatures were pooled and purified with the NucleoSpin® Extract II kit (Macherey-Nagel). In each of the genetic libraries, similar amounts of purified PCR product were cloned using the StrataClone PCR cloning kit (Stratagene) according to the manufacturer’s recommendations. Putatively positive transformants were screened by colony PCR using the vector-specific primers T3 and T7. Cells containing inserts of proper lengths were transferred to LB medium containing 96-well plates and sent to the sequencing service (LGC Genomics, Berlin) for plasmid preparation and Sanger sequencing with the primer Euk528F.

### Phylogenetic Analysis

Each chromatogram received by the sequencing service was inspected. Unreliable sequence ends were trimmed and low-quality sequences were excluded from further analysis. Chimera detection and taxonomic affiliation were done using KeyDNATools (http://www.keydnatools.com/) and BLAST [77] searches. Representative 18S rRNA sequences of a single operational taxonomic unit (OTU) based on 99% similarity were searched by creating a distance matrix and clustering the sequences according to the average-neighbor method in Mothur [78]. Multiple alignments were done using MAFFT version 6 [79] and poorly aligned positions were eliminated by Gblocks [80]. The alignments were inspected and manually corrected in BioEdit [81]. Maximum-likelihood phylogenetic trees with complete and partial 18S rRNA sequences were calculated using RAxML version 7.0.4 [82], which was run at the freely available Bioportal of the University of Oslo [83]. Both, tree construction and bootstrap analysis were done in 1,000 replicates on random starting trees under the evolutionary model GTRGAMMA. A consensus tree, displaying the bootstrap values, was computed in MrBayes [84] and the values were transferred into the topology tree having the best likelihood of 1,000 replicates. Sequences representing OTUs based on 99% similarity were deposited in GenBank under accession numbers JQ782278–JQ782384.

### Novelty Analysis

The novelty of the clonal sequences was analyzed as described by Massana et al. 2010 [85]. Briefly, each sequence was subjected to BLAST searches to extract the closest similarity to cultured organisms and to the closest environmental representative in GenBank. Both similarity values of each clone and the calculated average similarities of all t_0_ and t_end_ clones were displayed in a scatter plot indicating the similarity to the closest environmental representative on the x-axis and the similarity to the closest cultured representative on the y-axis.

## Supporting Information

Figure S1 **Similarity of samples before and after incubation based on presence and absence of DGGE bands.** Two-dimensional representation of a nonmetric multidimensional scaling plot based on the binary DGGE matrix for the three experiments (Exp1 in black, Exp2 in dark gray, Exp3 in light gray). Squares and triangles refer to t_0_ and t_end_ samples, respectively. Open and filled symbols represent unfiltered and 3-µm filtered samples, respectively. Note that the dark gray filled triangle represents the identical triplicate samples of Exp2.
(TIF)

Figure S2 **Phylogenetic affiliation of clones within picobiliphytes.** Maximum likelihood phylogenetic tree constructed with 32 partial and complete picobiliphyte sequences (241 informative positions). Clades follow the notation of Cuvelier et al. [52]. For further description see legend of Figure 6.
(TIF)
