# Supplementary figures and images for: Unveiling Trophic Functions of Uncultured Protist Taxa by Incubation Experiments in the Brackish Baltic Sea
Source: PLoS One. 2012 Jul 30;7(7):e41970. doi: 10.1371/journal.pone.0041970 (PMC3408427; doi:10.1371/journal.pone.0041970)

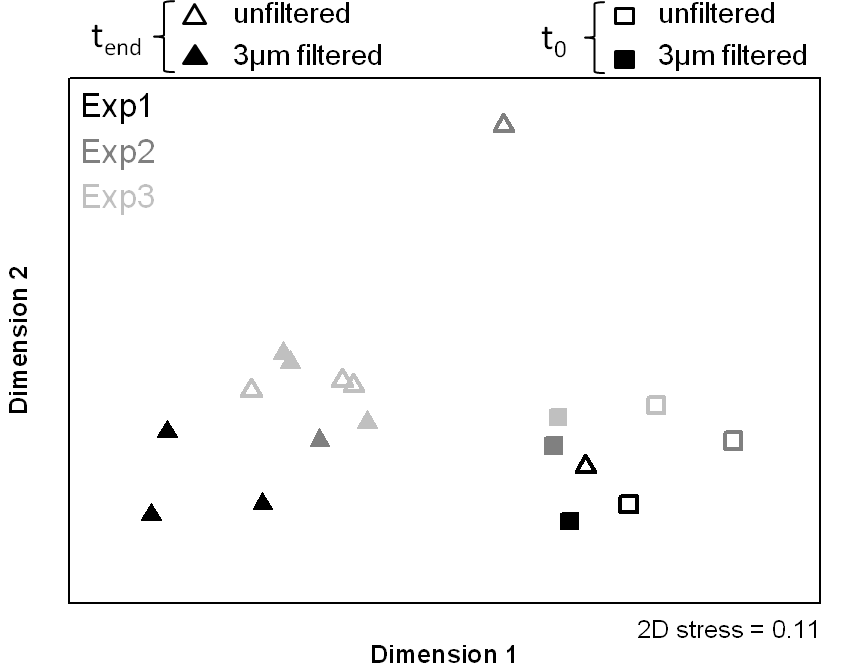

Supplement: Figure S1 — Similarity of samples before and after incubation based on presence and absence of DGGE bands. Two-dimensional representation of a nonmetric multidimensional scaling plot based on the binary DGGE matrix for the three experiments (Exp1 in black, Exp2 in dark gray, Exp3 in light gray). Squares and triangles refer to t0 and tend samples, respectively. Open and filled symbols represent unfiltered and 3-µm filtered samples, respectively. Note that the dark gray filled triangle represents the identical triplicate samples of Exp2. (TIF) [file pone.0041970.s001.tif]

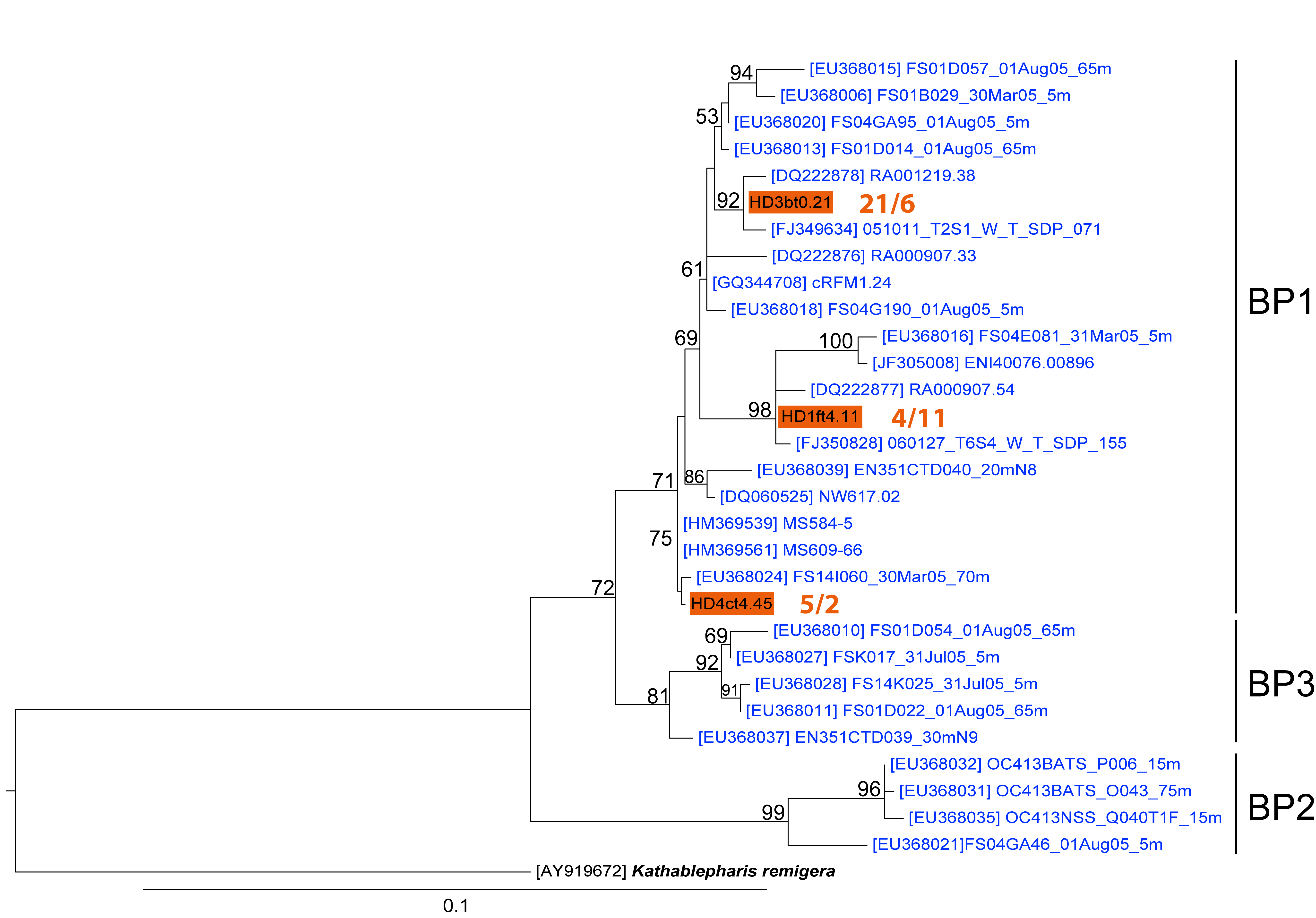

Supplement: Figure S2 — Phylogenetic affiliation of clones within picobiliphytes. Maximum likelihood phylogenetic tree constructed with 32 partial and complete picobiliphyte sequences (241 informative positions). Clades follow the notation of Cuvelier et al. [52]. For further description see legend of Figure 6. (TIF) [file pone.0041970.s002.tif]
